# RNA processing body (P-body) dynamics in mesophyll protoplasts re-initiating cell division

**DOI:** 10.1007/s00709-016-1053-0

**Published:** 2016-12-07

**Authors:** Dilbag S. Bhullar, Michael B. Sheahan, Ray J Rose

**Affiliations:** School of Environmental and Life Sciences, The University of Newcastle, Callaghan, NSW Australia

**Keywords:** Dedifferentiation, P-bodies, Protoplast division, RNA decapping, RNA degredation, RNA storage

## Abstract

The ability of plants to regenerate lies in the capacity of differentiated cells to reprogram and re-enter the cell cycle. Reprogramming of cells requires changes in chromatin organisation and gene expression. However, there has been less focus on changes at the post transcription level. We have investigated P-bodies, sites of post transcriptional gene regulation, in plant cell reprogramming in cultured mesophyll protoplasts; by using a YFP-VARICOSE (YFP-VCSc) translational fusion. We showed an early increase in P-body number and volume, followed by a decline, then a subsequent continued increase in P-body number and volume as cell division was initiated and cell proliferation continued. We infer that plant P-bodies have a role to play in reprogramming the mature cell and re-initiating the cell division cycle. The timing of the first phase is consistent with the degredation of messages no longer required, as the cell transits to the division state, and may also be linked to the stress response associated with division induction in cultured cells. The subsequent increase in P-body formation, with partitioning to the daughter cells during the division process, suggests a role in the cell cycle and its re-initiation in daughter cells. P-bodies were shown to be mobile in the cytoplasm and show actin-based motility which facilitates their post-transcriptional role and partitioning to daughter cells.

**Electronic supplementary material:**

The online version of this article (doi:10.1007/s00709-016-1053-0) contains supplementary material, which is available to authorized users.

## Introduction

Isolated mesophyll protoplasts are able to initiate division (Nagata and Takebe 1970) and ultimately produce mature plants via organogenesis (Takebe et al. 1971) or somatic embryogenesis (Lörz et al. 1977). Although the mechanisms involved in these processes are still not well understood, there is increasing understanding of the regulatory processes involved (Fehér 2015; Grafi and Barak 2015; Rose 2016), and high throughput studies have provided more information about the genes involved (Imin et al. 2004; Mantiri et al. 2008; Chupeau et al. 2013). The dedifferentiation and subsequent cell division appears to be a stress response which when linked to hormones in the culture medium leads to a reprogramming of the genome to initiate regeneration (Fehér 2015; Grafi and Barak 2015; Rose 2016).

In addition to investigations of gene regulation the organelle dynamics of chloroplasts, mitochondria and peroxisomes have been investigated in mesophyll protoplasts initiating division. Each organelle undergoes specific dynamics associated with dedifferentiation and the cell division induction processes. Chloroplasts dedifferentiate into proplastids and plastid division is initiated when the cells reach the numbers characteristic of meristematic cells (Thomas and Rose 1983). Mitochondria undergo massive fusion followed by proliferation (Sheahan et al. 2005) and there is massive proliferation of peroxisomes (Tiew et al. 2015). All three organelles are partitioned into fairly equal numbers, primarily dependent on the actin cytoskeleton (Sheahan et al. 2004a; Tiew et al. 2015).

P-bodies are specific ribonucleoprotein (RNP) granules that occur in the cytoplasm of eukaryotic cells and are involved in translational repression, mRNA decapping and degredation, and mRNA storage in plant cells (Xu et al. 2006; Jiao et al. 2008; Xu and Chua 2009). In plants these granules contain the decapping complex (Xu et al. 2006; Jiao et al. 2008; Xu and Chua 2009), including DECAPPING 1 and 2 (DCP1 and DCP2) and VARICOSE (VCS); as well as other proteins (Xu et al. 2006; Xu and Chua 2009; Gutierrez-Beltran et al. 2015). It is not known what mechanisms regulate decapping of selective mRNAs in P-bodies (Xu and Chua 2009). There are also other discrete cytoplasmic foci called stress granules which are involved in post-transcriptional gene regulation during stress (Gutierrez-Beltran et al. 2015). There may be exchange of mRNA and protein between P-bodies and stress granules (Anderson and Kedersha 2006).

New genes are transcribed during the dedifferentiation and genetic reprogramming process in the induction of cell division in cultured protoplasts. However, the expression of other genes and their transcripts are no longer required and their transcripts need to be degraded. Chupeau et al. (2013) have documented the up- and down-regulation of gene expression in cultured Arabidopsis protopasts and concluded that most dedifferentiation and reprogramming events occur within the first day of culture. As one approach to this process, we have investigated P-body dynamics. The results obtained show two phases of increase in P-body numbers. The timing of the first phase is consistent with the removal of messages no longer required as the cell transits to the division state, but may also be linked to the stress response associated with division induction in cultured cells. There is then another peak of P-body formation with partitioning of these P- bodies to the daughter cells during the division process. The partitioning may be due to the actin cytoskeleton dependent movement of P-bodies. This is consistent with the previously reported studies of Steffens and co-workers on P-body movement and the cytoskeleton (Steffens et al. 2014). P-body transmission may be important in ensuring continuity of the new cell division state.

## Results

### Visualising P-bodies

**Fig. 1:**
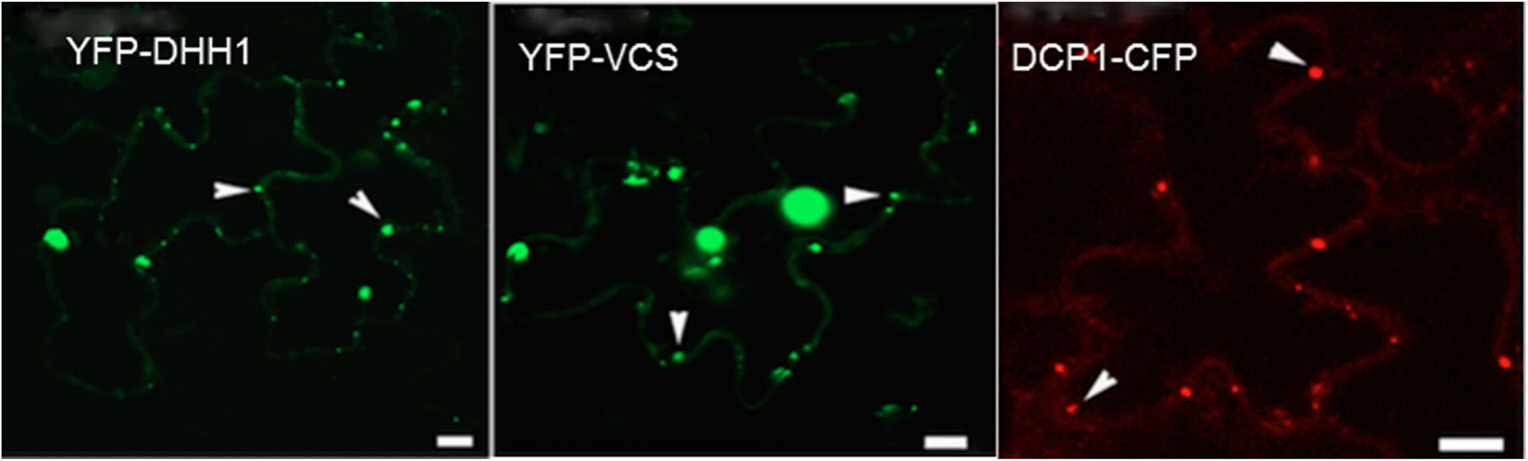
Subcellular localisation of DHH1, VCSc and DCP1 in tobacco epidermal leaf cells. Proteins are labelled with YFP (VCSc and DHH1; pseudo-coloured green) or CFP (DCP1; pseudo-coloured red). White arrows indicate P-bodies. P-bodies could be visualised as bright cytoplasmic dots in YFP-DHH1, YFP-VCSc and DCP1- CFP. Single focal plane images are shown. Bars =20 μm

In order to identify P-bodies we utilised a number of established markers; DHH1 (DEAD BOX HELICASE), VCS, DCP1 and DCP2 that have been used reliably in plants (Xu et al. 2006; Xu and Chua 2011). The subcellular localisation of YFP- DHH1, YFP-VCSc and DCP1-CFP was assessed by using agroinfiltration of tobacco leaves. All three markers consistently localised to discrete cytoplasmic foci in leaf epidermal cells (Fig.1).

**Fig. 2:**
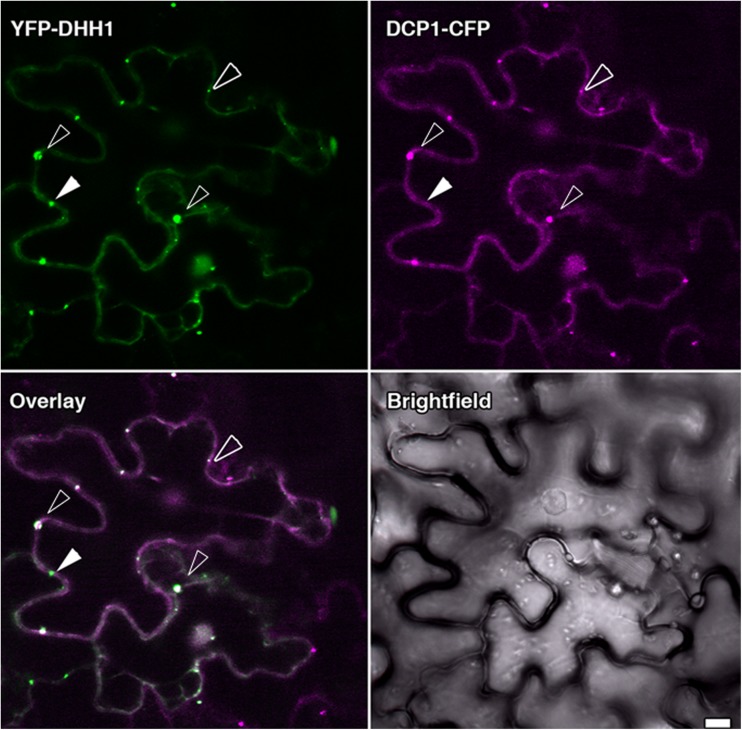
Co-localization analysis of YFP-DHH1 with DCP1- CFP. DCP1- CFP was co-expressed with YFP-DHH1 in tobacco epidermal leaf cells. Blank arrowheads indicate the cytoplasmic foci marked by both proteins. While DCP1 frequently localised to cytoplasmic foci marked by DHH1, at times DHH1 marked P-bodies devoid of DCP1 were observed as well. Solid white arrows indicate cytoplasmic foci marked by DHH1 lacking DCP1. Bars =10 μm

To examine if the three markers localised to the same cytoplasmic foci, co-expression studies were carried out. Co-expression of YFP-DHH1 and DCP1-CFP confirmed that both proteins could localise to the same cytoplasmic foci (Fig. 2a-d). Similar results were observed with YFP-VCSc and DCP1-CFP (Fig. S1). This indicated that DHH1 and DCP1, VCS and DCP1 resided in the same complexes. However, from the merged images it was apparent that not all visualised foci contained all of the markers, possibly reflecting their dynamic nature. Consistent with this were our results with DCP2-CFP localisation, which only was observed when co-expressed with VCS in tobacco protoplasts (Fig. S2).

**Fig. 3:**
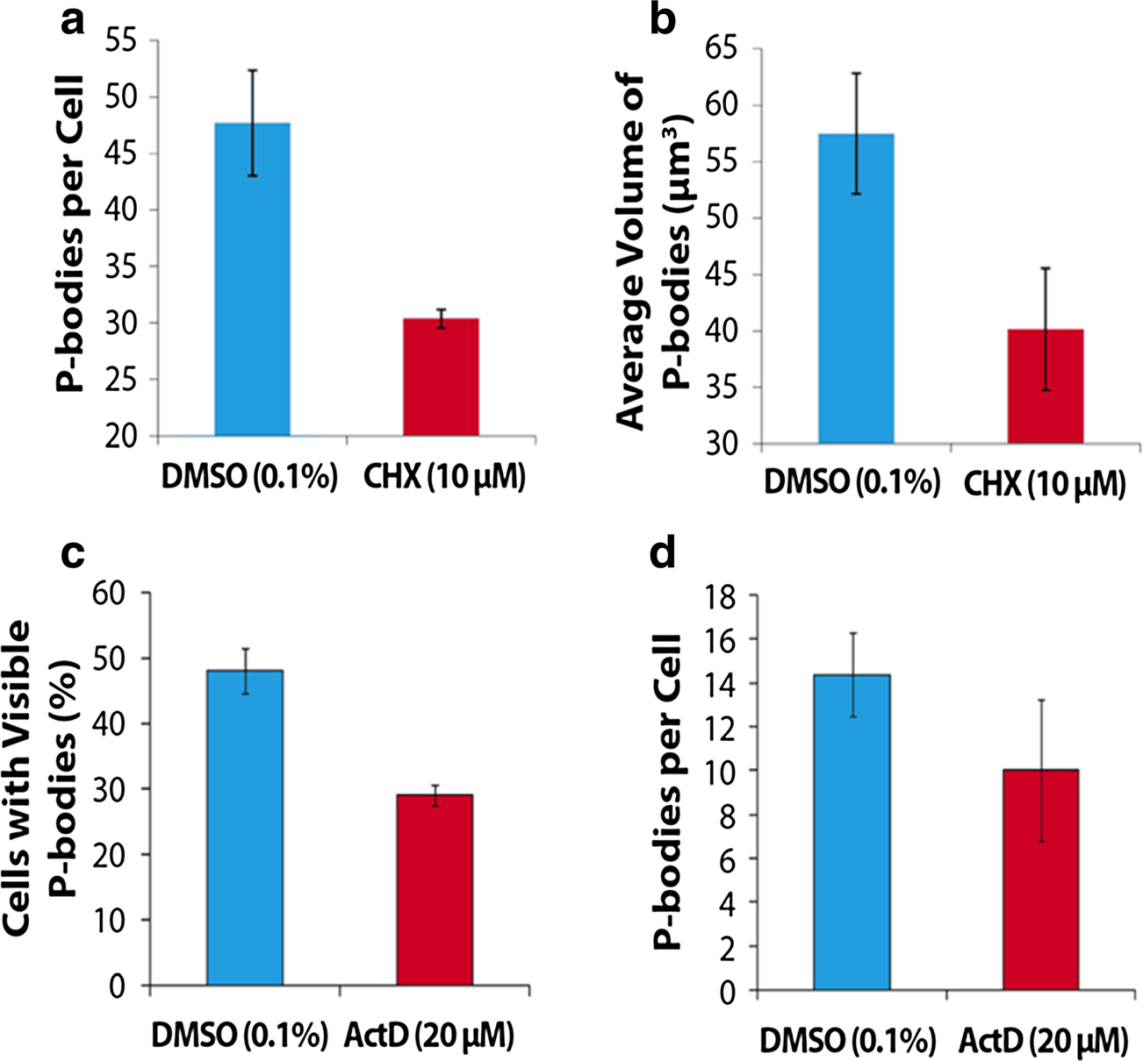
Quantitative analysis of P-body dynamics in CHX and ActD treated protoplasts. (a) P-body number per cell was significantly reduced by 10 μM CHX, *p* = 0.008, and (b) volume was reduced, *p* = 0.081. (c) In samples treated with 20 μM ActD a significant reduction in the number of cells with visible P-bodies was observed, *p* = 0.010, and (d) there was a smaller reduction in P-body numbers, *p* = 0.380. A students’ t-test was applied to each data set and *p*-values are given. Protoplasts were from transiently transformed leaves and the marker fusion protein was YFP-VCSc

To obtain additional evidence that the cytoplasmic foci were P-bodies, tobacco mesophyll protoplasts expressing 35S–YFP-VCSc were treated with the inhibitors cycloheximide (CHX) and Actinomycin D (ActD). CHX inhibits translation by binding to the E-site of the 60S ribosome subunit, blocking tRNA translocation and consequently translation elongation (Schneider-Poetsch et al. 2010). This results in a stalling of mRNA on polysomes and renders mRNA unable to exit translation machinery (Eulalio et al. 2007). Treating cells with CHX (10 μM) for 1 h caused a significant decrease in the number, and a substantive reduction in total volume, of P-bodies per cell (Fig. 3a and b) as no mRNA is available for decapping. This effect of CHX is well established for P-bodies (Xu et al. 2006; Parker and Sheth 2007, Goeres et al. 2007; Weber et al. 2008; Maldonado-Bonilla 2014). ActD inhibits transcription by binding to DNA within the transcriptional complex and halting the elongation of growing RNA chains (Sobell 1985). Existing mRNAs in the presence of ActD are freely translateable but there is less new mRNA. Treating protoplasts with ActD (20 μM) for 3 h (ActD is less permeable to plant cells than CHX e.g. De Varennes et al. 1985) resulted in a significant reduction in the number of cells expressing P-bodies (Fig. 3c) but only a small reduction in the number of P-bodies per cell with large variation (Fig. 3d). P - body formation upon ActD treatment is highly variable due to differences in ActD uptake or decay rates in individual protoplasts.

### P-body dynamics during mesophyll protoplast dedifferentiation and cell proliferation

**Fig. 4:**
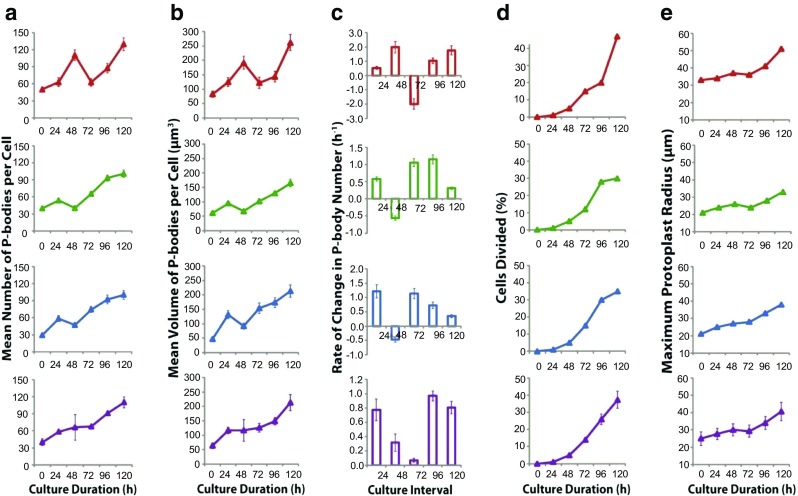
P-body dynamics during mesophyll protoplast dedifferentiation and re-entry into the cell cycle. Replicate one shown in red, two in green, three in blue and the average in purple. (a) The number of P-bodies observed in cultured mesophyll protoplasts. Note that all replicates show a similar trend, that is two phases of P-body proliferation, however, replicates two and three are shifted to earlier culture changes relative to replicate one, leading to a masking of the biphasic proliferation trend in the averaged results (purple trace). (b) Total volume of P-bodies in a cell. Similar trend as to that shown in (a) and consistent with little change in the average size of P-bodies over the culture interval. (c) Rates of change in P-body number. Note that on average (purple graph), the biphasic burst of P-body proliferation is clearly evident. (d) Proportion of cells divided. On average (purple trace), cell division increased linearly after 48 h culture. Note that the increase in P-body number appears to correlate with the amount of cell division. (e) The maximum protoplasts radius, an indicator of cell volume and therefore cell expansion during culture. Error bars represent SE. Protoplasts were isolated from tobacco leaves from transgenic plants expressing YFC-VCSc

With evidence that P-bodies could be reliably visualised we investigated P-body dynamics in cultured tobacco mesophyll protoplasts using 35S–YFP-VCSc. The number of P-bodies observed during the progression of cultured mesophyll protoplasts is shown in Fig. 4. The three biological replicates are presented individually as biological factors such as developmental stage of the leaves used for protoplast isolation and the microenvironment affect the progression of the cultures. The results showed clear trends related to the developmental stage. The first phase of increase in P-body number appeared to correspond to dedifferentiation (0–48 h), where a significant increase in P-body number was followed by a decrease preceding cell cycle re-entry. The second rise in P-body number correlated with cell proliferation.

Analysis of the rate of change in P-body number in replicate one revealed a high rate of increase in P-body number from 24 to 48 h followed by a reduction in the 48–72 h culture interval (Fig. 4a, red trace). Similarly, although occurring earlier, in replicates two and three P- body number increased from 0 to 24 h followed by a reduction in the 24–48 h period. On average, a high rate of increase in P-body number in the first 24 h was followed by a decrease in the rate of increase in the 24–48 h culture interval. If these data are plotted as rate changes (Fig. 4c) these latter changes are readily visualised. The changes in total P-body volume per cell parallel the change in P-body number (Fig. 4b).

We expected an increase in P-bodies as a result of dedifferentiation, that is an initial increase as mRNAs accumulate in P-bodies for degredation then a decline as degredation is completed. This occurs in response to a need to remove unnecessary transcripts related to the mesophyll cell fate. What was surprising was that following the earl peak of P-body formation and subsequent decline, P-body numbers increased again as cells entered cell proliferation. It is evident from the data that numerous P-bodies are present in rapidly proliferating cells.

In the first replicate, a small increase in cell division was observed from 72 to 96 h followed by much greater increases in cell division from 96 to 120 h. P-body number correlated with this change, with a small increase in number from 72 to 96 h, followed by a further increase in number from 96 to 120 h (compare Fig. 4a and d; red traces). In replicates two and three, the cell division rate increased from 72 to 96 h and significant changes in P-body number were observed, with numerous P-bodies at 96 h. The number of cells divided between 96 and 120 h did not change greatly, which was reflected in a similar marginal increase in P-body number, (compare Fig. 4a and d; green and blue traces). It is evident from the data that numerous P-bodies are present in rapidly proliferating cells. There is also a small upward trend in mean protoplast radius (Fig. 4e). The onset of protoplast division is imperfectly synchronised, so as protoplasts increase in size and then divide the net effect is an upward trend in size until there is continued cell cycling of the population.

**Fig. 5:**
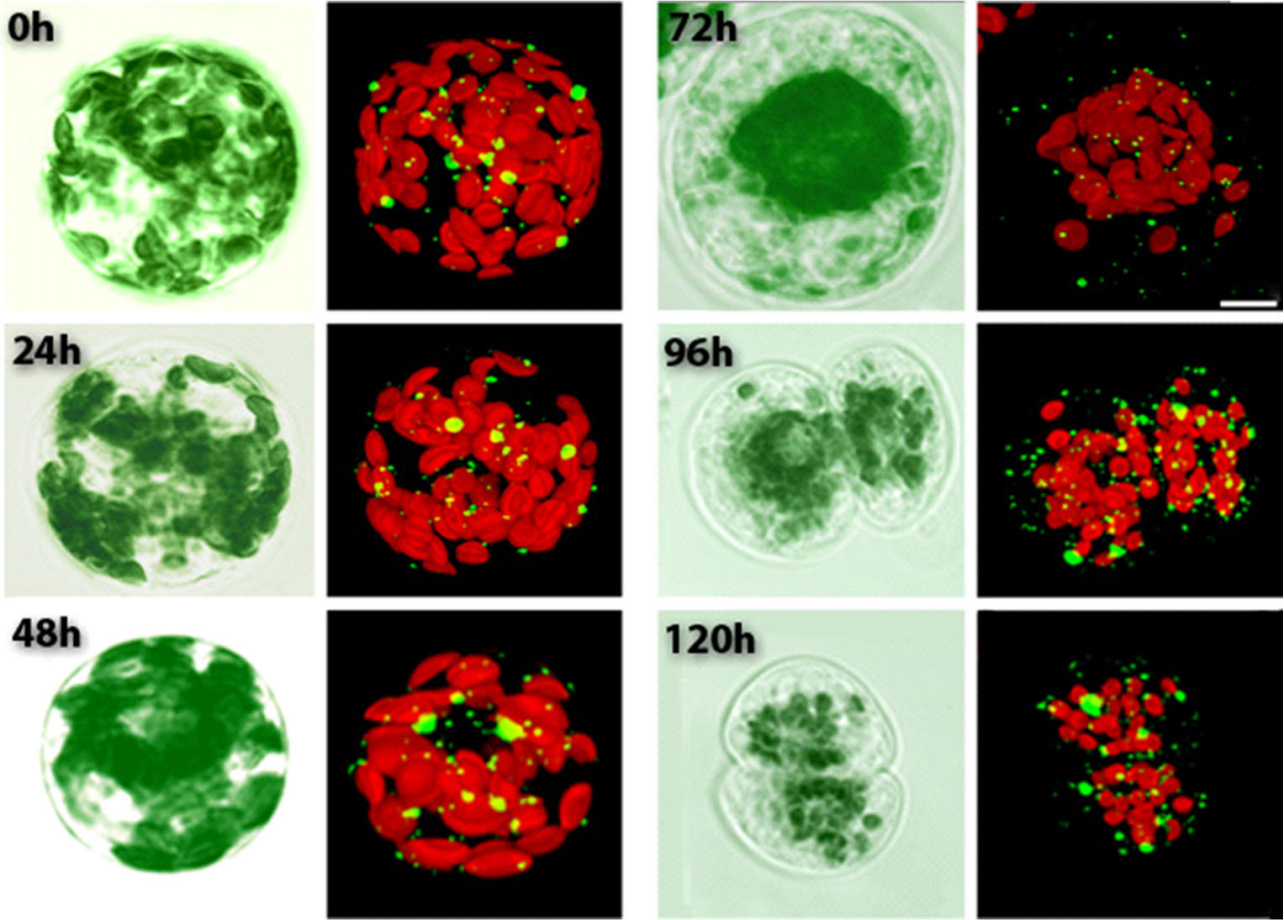
Visual observations of cultured protoplasts. Protoplasts isolated from transgenic tobacco leaves expressing YFC-VCSc observed over 120 h. Images taken at 24 h time intervals from 0 h (isolation time point) to 120 h (cultured protoplasts). Simultaneous visualisation of autofluoroscent chloroplasts provides insights into cell division pattern. Chloroplasts (red) are distributed across the entire cell area (0–48 h), however they cluster around the nucleus (72 h) before the onset of cell division, as earlier described (Sheahan et al. 2004a). Cell division shown at 96 h and 120 h. P-bodies (small green spheres) can be observed distributed throughout the cell with no particular localization. It appears that P-bodies are increasing in number as the culture progresses (0–120 h). The images of representative protoplasts at the different time points show a general trend of cell division, increased P-body number and chloroplast clustering. Bar: 10 μm

The conclusions based on cell number changes are supported by the visualisation of changes in typical protoplasts (Fig. 5). At the time the cell was in division (Fig. 5, 96 h) there were clear increases in P-body numbers. It is suggestive that P-bodies increase in numbers prior to division and then approximately half are transmitted to each daughter cell. What is clear is that irrespective of division mechanisms, the P-bodies are transmitted to the next generation of cells. We do not know if at this stage DCP2, the catalytic subunit of the decapping complex, is absent in the transmitted P-bodies so that stored mRNA can be translated. The DCP2 localisation and co-localisation visualisation studies do suggest that DCP2 recruitment is dynamic, as occurs in germination where DCP2 is absent in the seed and recruited upon germination (Xu and Chua 2009).

### Movement of P-bodies

**Fig. 6:**
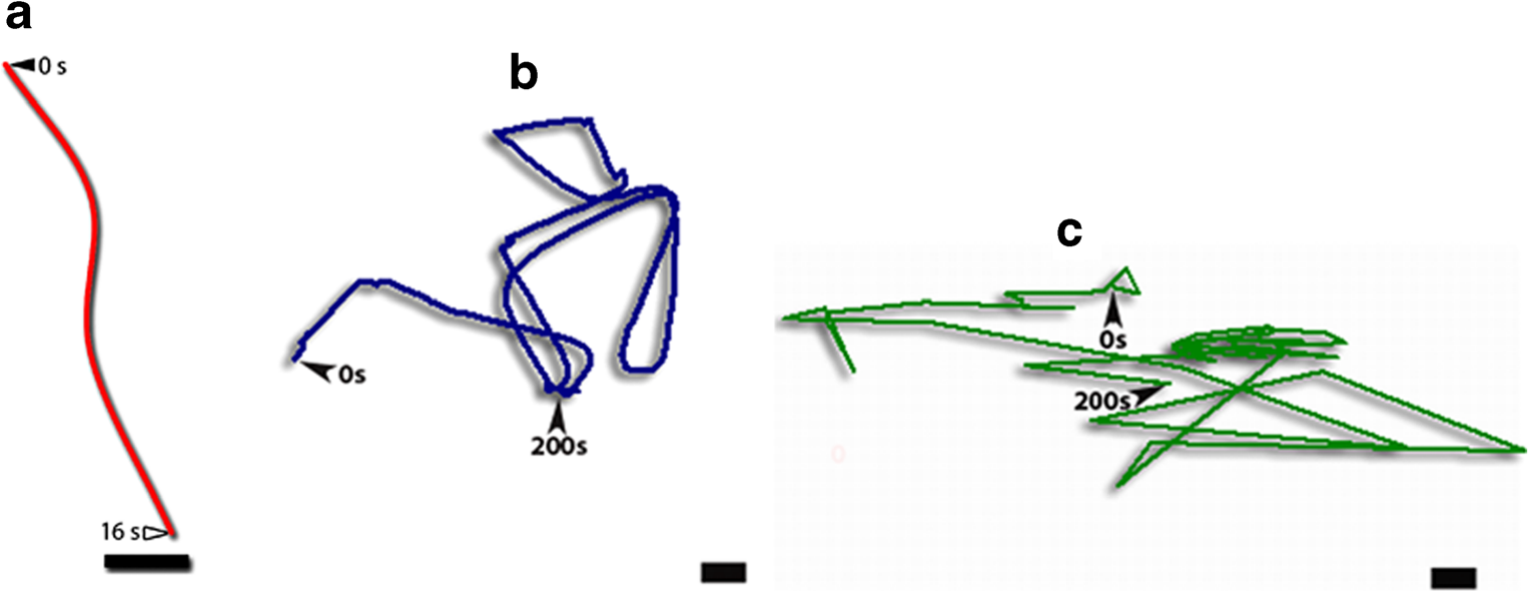
Tracking of P-body movements showing motion paths of P-bodies. Transiently transformed tobacco epidermal leaf cells were visualised for 200 s. P-bodies generally exhibited erratic and circular motion (a,b) while occasional rapid vectorial movements were also observed (c). Bars =10 μm

Given that the P-bodies are transmitted to daughter cells, it raises the question of how they are distributed in the cell and the movements they undergo, that might give a basis for partitioning. We analysed P-body movements in mesophyll cells of tobacco leaf discs using time-lapse fluorescence microscopy (Supplementary Video 1, Fig. 6). Most P-bodies exhibited erratic movements that were somewhat circular, did not lead to significant displacement but did cover a substantial area of cytosol. Although less frequent, some P-bodies travelled relatively long distances (up to 22 μm) in a rapid vectorial manner, with instantaneous velocities up to 24 μm.s^−1^. This latter observation suggested the cytoskeleton could be employed in some movements. To investigate the role of the cytoskeleton in P-body movement, we disrupted actin filaments (AFs) with Latrunculin B (LatB) or microtubules (MTs) with oryzalin (Ory) and examined P-body motility.

**Fig. 7:**
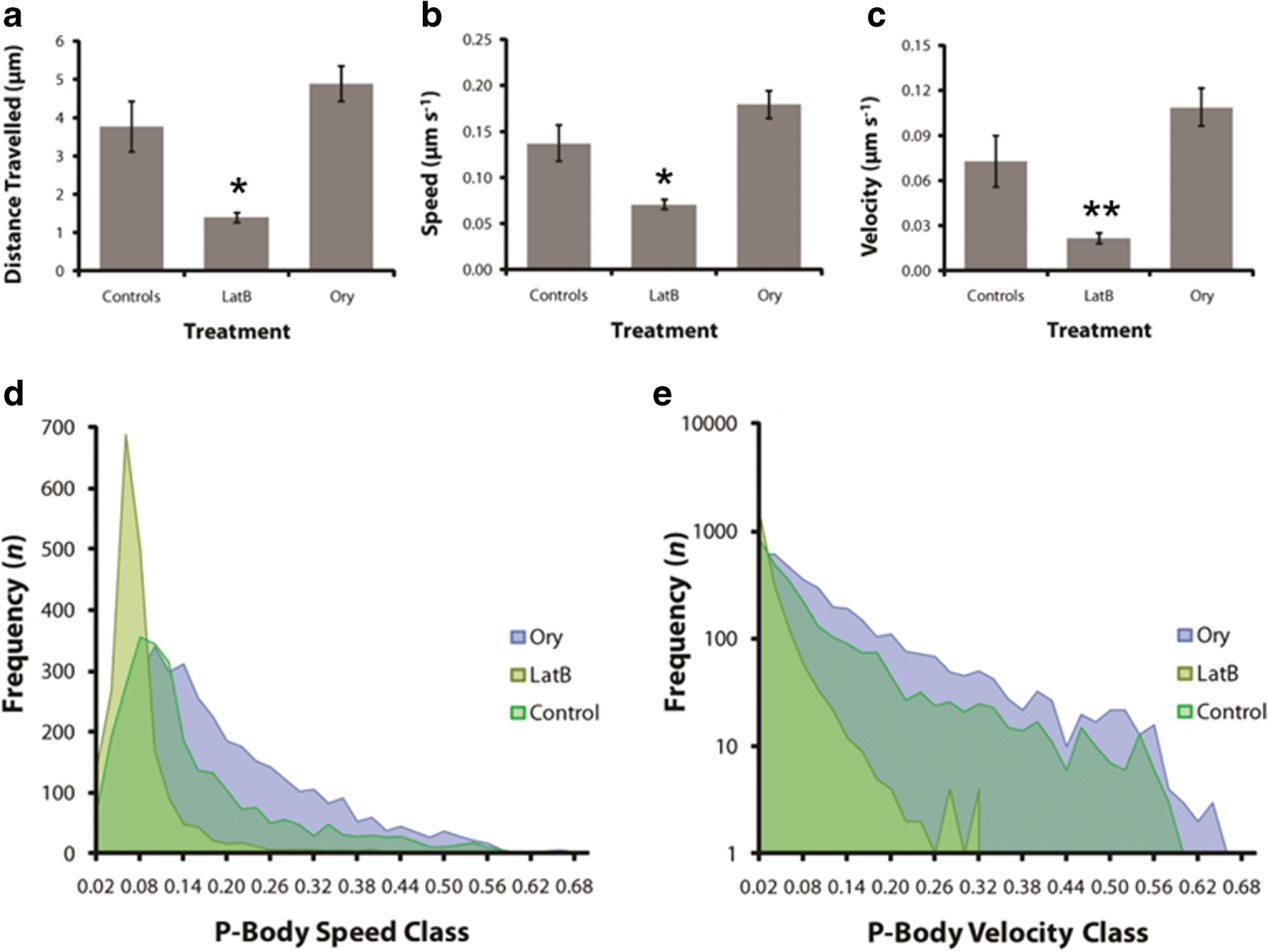
(a-c) Means of five replicates with SE for time-lapse image series depicting the effects of LatB and Ory on P-body motility (a), speed (b) and velocity (c) in tobacco epidermal leaf cells. LatB treatment reduced vectorial movements of P-bodies, best represented by the velocity analysis (c), while speed was also reduced to a lesser extent (b). (d-e) Frequency area graphs summarising the motile characteristics for the whole population of P-bodies analysed in each treatment. Most P-bodies exhibited lower speeds (d) and velocities (e) consistent with a predominantly erratic motility. Treatment with LatB however, skewed the results toward the left of the graph with substantially lower speeds, and more significantly, much lower velocities. Note that (e) has a logarithmic ordinate axis. Speed is distance/unit time (μm s^−1^) while velocity is displacement/unit time (μm s^−1^). P-bodies and AFs were visualised simultaneously in tobacco epidermal cells co-transfected with YFP-VCSc (P-body marker) and GFP-faBD2 (AF label). Standard errors indicated and ^*^ *p* < 0.05, ^**^ *p* < 0.01 for a, b and c LatB versus control. Ory in all cases not significantly different to control

Treating leaf discs with LatB significantly reduced P-body movement within cells, with a striking inhibition of vectorial movement and in many cases even Brownian type motions were inhibited (Fig. 7a-e). Quantitative analysis confirmed that LatB-treatment halved the average speed of P-bodies relative to controls while velocity was reduced more than 70%. In comparison to control cells, P-body movements in LatB treated cells were more confined and constricted (Fig. 7a-e). In contrast, disrupting MTs had no observable impact on P-body motility, with quantitative analysis indicating that though Ory-treatment caused an increase in P-body speed (29%) and velocity (57%) relative to controls it was not significantly different to controls (Figs 7a-c). These data indicate that MTs have little role in promoting the motility of P-bodies, rather MTs tend to constrain P-body movement.

### P-body and actin co-localisation

**Fig. 8:**
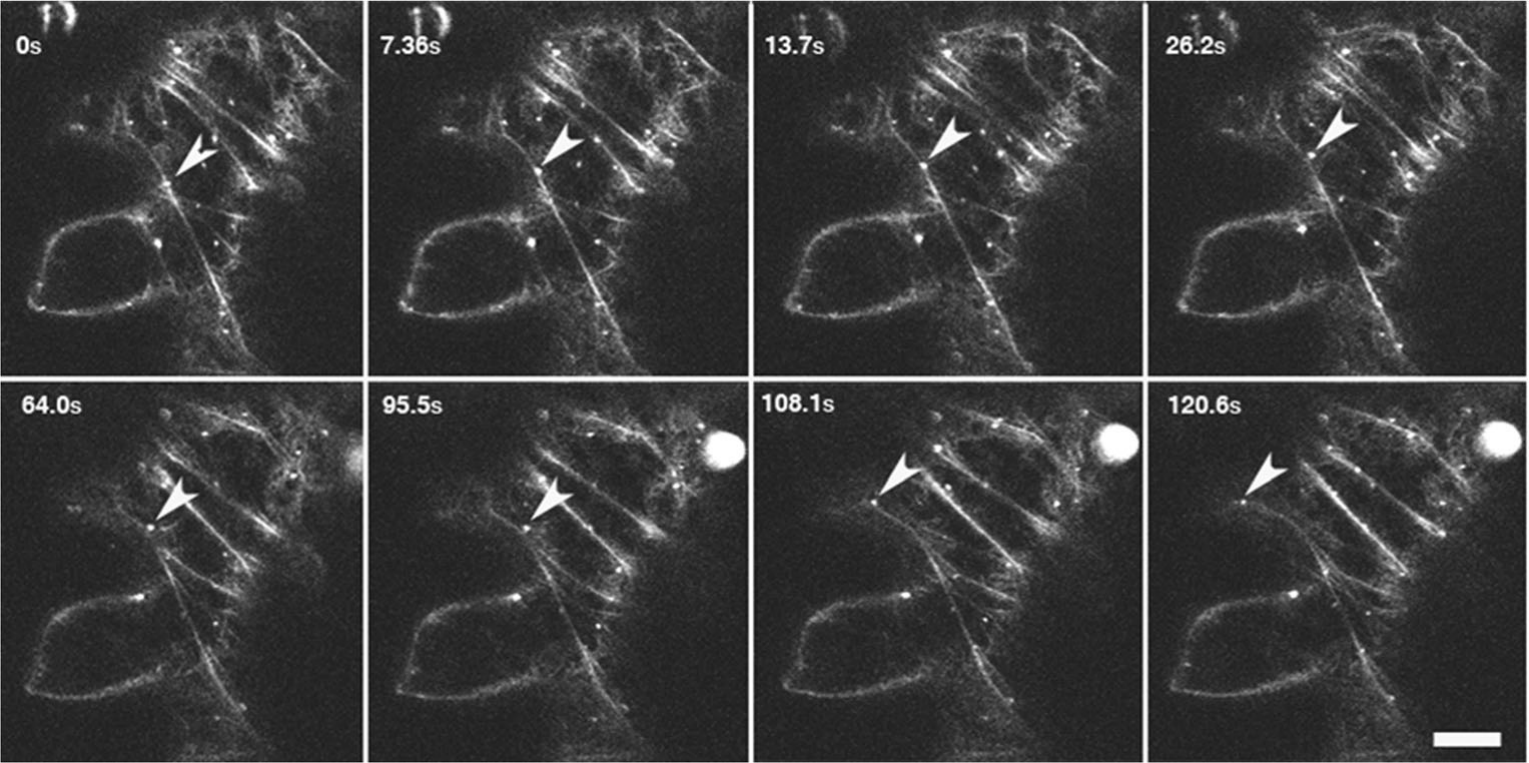
P-body actin colocalisation. The actin cytoskeleton can be observed as fine filaments and P-bodies as round white spheres in tobacco epidermal leaf cells. Images series was taken over a period of 2 min. Arrows represent a P-body that appears to travel on an AF bundle. P-bodies and AFs were visualised simultaneously in tobacco epidermal cells co-transfected with YFP-VCSc (P-body marker) and GFP-faBD2 (AF label)

Our results clearly indicate a role for the actin cytoskeleton in the more substantive (non-erratic) movement of P- bodies. To gain insight into the potential mechanism for this AF-dependent motility, we visualised P-bodies and AFs simultaneously in tobacco leaf epidermal cells co-transfected with YFP-VCSc (P-body marker) and GFP-fABD2 (AF label). While both AFs and P-bodies were visible in the same confocal detection channel due to the overlap of GFP and YFP excitation and emission profiles, we were able to distinguish P-bodies from the actin cytoskeleton on the basis of morphological discrimination, whereby P-bodies were observed as spheres and AFs as filamentous networks and bundled structures (Video 2 and Fig. 8). Analysing a series of time-lapse images, we found that a small proportion of P-bodies appeared to be directly aligned with AFs and movement of the P-bodies along the AFs. However, in many instances we observed P-body motility, albeit largely erratic in nature, occurring with no clear interaction with AFs in the vicinity of the P-body.

## Discussion

### P-body visualisation

To identify P-bodies in this study we used translational fusions that had been developed and used by Xu et al. (2006). These marker translational fusions, DCP1, DCP2, DHH1 and VCSc fused with either CFP or YFP, are involved with the 5′ decapping complex (Xu et al. 2006; Maldonado-Bonilla 2014). One model put forward by Xu and Chua (2011) has DCP1, DCP5 and possibly DHH1 involved in formation of RNPs for translational repression of mRNAs. DCP2 and VCS are then recruited for decapping. The data we obtained are generally consistent with this. We localised DCP1, DHH1 and VCS to cytoplasmic foci both individually and in co-expression studies. The localisation of DCP2 in P-bodies was only possible by co-expression of YFP-VCSc. It is possible that the level of VCS in our tissue was not optimum for DCP2 recruitment (e.g. Iwasaki et al. 2007). VCS is central to the assembly of P-bodies (Goeres et al. 2007) as the *vcs* mutant fails to accumulate DCP2-CFP into cytoplasmic foci.

To further check that we had visualised P-bodies we investigated the effect of CHX and ActD on P-body assembly using the VCS marker. As previously shown (Xu et al. 2006; Parker and Sheth 2007, Goeres et al. 2007; Weber et al. 2008; Maldonado-Bonilla 2014) when mRNA is trapped on the translation machinery by CHX then P-bodies cannot access cytoplasmic mRNA. ActD, which is also known to inhibit P-body formation (Aizer et al. 2008) had a lesser effect on P-body numbers as it does not eliminate mRNAs already present. The markers we used are good tools to track P-bodies in plant cells.

### P- bodies in protoplasts dedifferentiating and initiating cell division

When mesophyll protoplasts are re-programmed into cell division, some pre-existing messages characteristic of the mesophyll cell would be expected to be degraded as the cell prepared to have a new gene expression pattern. Consistent with the degredation of pre-existing mRNA was an increase in the number and size of the P-bodies following protoplast isolation and early culture. This is probably due to accumulation of pre-existing mRNAs in P-bodies and their subsequent 5′-decapping and degredation by the 5′-3’exoribonuclease XRN4 (Xu et al. 2006). XRN4 is known to occur in P-bodies and *xrn4–5* mutants cause accumulation of mRNA in p-bodies (Weber et al. 2008). Some concomitant changes in mRNA storage and translation re-initiation cannot be ruled out (Brengues et al. 2005). There is also a possible connection to stress as there are increases in oxidative stress-related reactive oxygen species (ROS) early on in culture (Tiew et al. 2015). P-bodies, though likely to respond to stress and interact with stress granules, are distinct from them because of their different components (Weber et al. 2008; Gutierrez-Beltran et al. 2015). Thus the increase in P-body number, and volume, could be driven by mRNA degredation, but with an additional stress component. Stress granule analysis would be beneficial in providing a more comprehensive understanding of post-transcriptional regulation in early protoplast culture.

Following the rise in P-body numbers there is a decline, followed by further increases as cells enter cell division and continue cell proliferation. It is possible this is because of mRNA degredation that is directly related to regulatory mechanisms in cells entering division. However, it is also known that P-bodies can enable translational arrest of mRNA and its subsequent utilisation as well as mRNA degredation (Brengues et al. 2005; Balagopal and Parker 2009; Xu and Chua 2011; Maldonado-Bonilla 2014), and P-bodies could be potential reservoirs for mRNA information for new daughter cells entering the cell cycle. If this was the case, then it would be expected that the P-bodies would be transmitted to the next generation, and this did occur (Fig. 5). In Arabidopsis, single mutant alleles of DCP1, DCP2 and VCS are lethal at the seedling stage with phenotypes including vein, leaf blade and root defects (Xu and Chua 2011). These developmental effects do not necessarily mean a requirement for transmission to daughter cells. It would nevertheless seem valuable to initiate subsequent cell division cycles by having key messages to set in train the next cycle. In human HeLa cells (Yang et al. 2004), P-bodies were observed to increase in size through the cell cycle and then most disassembled prior to mitosis, reforming in G1. However there were larger and more numerous P-bodies in proliferating as opposed to quiescent cells. In yeast, transmission to daughter cells has been demonstrated (Garmendia-Torres et al. 2014) and was found to play a very important role in subsequent growth of daughter cells under nutrient limiting conditions. However, if a cell did not receive P-bodies it was not fatal and new P-bodies formed. It would seem that P-bodies are important in an optimum cell cycle with a possibility of additional significance under stress conditions where there is an advantage if transmission does occur. Biologically, it is well documented that maternal RNA germ granules are transmitted from the oocyte to the embryo to facilitate the next generation in Drosophila, *C. elegans*, Xenopus and zebra fish (Voronina et al. 2011). Germ granules share components with P-bodies (Gallo et al. 2010; Voronina et al. 2011). These germ granules are partitioned in early cell divisions (Gallo et al. 2010) and have been related to cell fate (Voronina et al. 2011) and also to stress (Gallo et al. 2010). In the plant life cycle seeds accumulate translationally arrested mRNAs in P-bodies that have a subsequent role in early germination (Xu and Chua 2009).

In yeast, when P-bodies are transmitted to daughter cells the P-bodies are transported to the bud site and is actomyosin dependent (Garmendia-Torres et al. 2014). Our studies indicate that P-bodies can move along AFs, but not all P-bodies associate with the cytoskeleton. The situation in plants contrasts with animal cells where P-body motility is microtubule - based (Aizer et al. 2008). Previous plant studies carried out in Arabidopsis have shown AF-based movement. Using seedling shoot epidermal cells, Hamada et al. (2012) have shown AF-dependent transport of P-bodies which frequently pause at cortical microtubules. However despite removing MTs with Ory, P-bodies still paused. Our results are consistent with those of Hamada et al. (2012). P-body pausing may be a reflection of direct actin – organelle interaction albeit closely associated with but not dependent on microtubules. Steffens et al. (2014) found that P-body motility in plants is governed by myosin XI members binding to AtDCP1, to mediate actin-based motility. YFP-VSCc and DCP1 foci can overlap when localised to the P-bodies as shown here (Fig. S1) and by Xu et al. (2006). This likely occurs by an interaction with VSCc, VSC and DCP1 (Xu et al. 2006). The movements observed in our study, including those directly involving the cytoskeleton, enable the granules to be distributed throughout the cytoplasm which would facilitate their post-transcriptional role. This would also facilitate the fairly equal partitioning to daughter cells we observed. It is also feasible, as indicated above, that there are roles for the P-body mRNAs in the next cell cycle. Potentially this could be in relation to chromatin and/or cytoplasmic organelle organisation.

What our data collectively emphasise is that the P-bodies and their involvement in post transcriptional regulation likely have an important role in the induction of the cell cycle and continuous cell proliferation and are worthy of further investigation in this context, and the cell cycle more generally.

## Methods

### Plant material

For transient expression assays (agroinfiltration), glasshouse grown tobacco (*Nicotiana tabacum* cv. Xanthi) plants were used. The glasshouse conditions were as described in Sheahan et al. 2004a. Axenic shoot cultures of stable tobacco transgenics were established as described by Potrykus and Shillito (1986) and Sheahan et al. (2004a) and used for leaf disc studies and protoplast formation. Seeds were first surface sterilised by washing with 70% (*v/v*) ethanol, followed by rinsing in 10% (*v/v*) commercial bleach (White King, Pental Products, Melbourne, Australia) for 15 min. Seeds were washed five times with sterile water before plating onto solid MS media (Murashige and Skoog 1962 with 1% [*w/v*] sucrose). Plates were placed in a culture room at 25 °C with 16 h photoperiod and lighting of 50 μmol m^−2^ s^−1^. Following seed germination and seedling growth for 1 month, seedlings were cut at the hypocotyl and transferred to 6.5 cm culture pots containing 50 mL of MS media (with 1% [*w/v*] sucrose). Shoot cultures were placed under the same conditions as for seedlings.

### Protoplast isolation and culture

Protoplasts were isolated using sterile techniques from either transiently transformed glasshouse grown tobacco leaves or axenic shoot cultures of stable tobacco transgenics according to methods similar to those previously described (Sheahan et al. 2004a). Leaf tissue cut into 0.5–1 cm^2^ sections was floated on 5 mL of protoplast enzyme solution in a 5.5-cm Petri dish. The protoplast enzyme solution consisted of 1.0 g cellulase ‘Onozuka’ RS (Yakult Pharmaceutical, Tokyo, Japan) and 1.0 g macerozyme R10 (Yakult Pharmaceutical), 1.0 mL 500 mM MES and 0.5 g BSA; made to 100 mL with NT medium (Thomas and Rose 1983) and pH adjusted to 5.6. The tissue was digested at 25 °C overnight in complete darkness. Protoplasts were purified by filtration through a sterile 55-μm nylon membrane (Sartorius Membrane Filter, Gottingen, Germany), into a 10-mL centrifuge tube. The filtrate was mixed with Percoll (GE Healthcare, Parramatta, Australia) to a final concentration of 20% (*v:v*) and 1.5 mL of MW5 salt solution (Thomas and Rose 1988) was carefully layered on top of the Percoll-filtrate mix. After centrifugation at 60 x g for 5 min in a swing-out rotor, floated protoplasts were collected at the MW5 interface with the Percoll-filtrate mix. Protoplasts were washed twice with MW5 at 80 x g for 5 min and finally resuspended in 2.2 mL of NT medium. Protoplasts were plated at a density of 7.5 × 10^4^ cells per mL in 0.6 mL of protoplast suspension per well in 25-well Sterilin® plates (Thermo Fisher, Waltham, MA, USA). Incubation was at 26 °C in low light (25 μmol m^−2^ s^−1^).

### Constructs for P-body and actin filament visualisation

Working constructs of YFP-DHH1, YFP-VCSc, DCP1-CFP and DCP2-CFP with expression driven by the Cauliflower Mosaic Virus 35S promoter (CaMV35S) in a pMBP binary vector were kindly provided by Dr. Nam-Hai Chua and Dr. Jun Xu (Rockefeller University, New York). In YFP-VCSc, only the C-terminal region of VCS comprising about 400 amino acids on the C-terminal is fused to YFP, since the C-terminal region of VCS is necessary and sufficient for its localization to P-bodies (Xu et al. 2006). In YFP-DHH1, DCP1-CFP and DCP2-CFP, the full length DNA sequence is fused to YFP or CFP encoding sequences on N-termini and C-termini respectively. The actin filaments (AFs) were labelled with the GFP-fABD2 construct of Sheahan et al. (2004b) in the binary vector pART27 (Sheahan et al. 2004b). The binary vectors were electroporated into the *Agrobacteriun* strain AGL1. DHHI is DEA(D/H)-box RNA helicase family protein AT3G61240; DCP1 is DECAPPING1 AT1G08370; DCP2 is DECAPPING2 AT5G13570; VCS is VARICOSE AT3G13300.

### Transient and stable transformation

Glasshouse grown *N. tabacum* was transiently transformed using the agroinfiltration method (Yang et al. 2000). Stable transformation of axenic shoot cultures of *N. tabacum* was by the leaf disc procedure of Horsch et al. 1985 as described in Sheahan et al. 2004a.

### Inhibitor treatments

Translation elongation was blocked by 10 μM CHX and transcription by 20 μM ActD. Both inhibitors were prepared as 1000X stocks in DMSO and stored at -20 °C. CHX (0.5 μL) or ActD (1 μL) was added to 0.5 mL of protoplast suspension. Protoplasts were mixed gently with a 3 mL transfer pipette to mix the drugs and then incubated for 1 h in CHX treatment or 3 h in ActD. In all experiments, 0.1% (*v*/v) DMSO was used as a control.

Inhibitor studies of AFs or MTs were carried out on transgenic *N. tabacum* leaf discs. AFs were disrupted by treatment with 1 μM Latrunculin B (LatB; Merck Millipore, Bayswater, VIC, Australia) and MTs by 10 μM oryzalin (Ory, Crescent Chemical Co., Islandia, NY, USA). The inhibitors were prepared in 0.1% (*v:v*) DMSO and the controls were incubated in 0.1% (*v:v*) DMSO. Leaf discs were pierced centrally by a needle and submerged in 5 mL tubes overnight with the solutions containing 0.02% (*v:v*) Silwet L.-77 detergent (Bio-world, Dublin, OH, USA). This ensured uniform uptake of the solutions based on tests with the fluorescent dye fluorescein diacetate.

### Confocal microscopy and image analysis

Leaf discs excised from transformed tissue and protoplasts isolated from transformed leaves were visualised by a confocal laser scanning microscope (CLSM; Zeiss LSM510). Leaf discs were mounted on a slide and a coverslip (50-mm, No.1; Marienfeld Products, Lauda-Königshofen, Germany) placed over the leaf discs. Protoplasts (120–150 μL) were loaded on to a welled-slide (No. 7103; Sail Brand, Trajan Scientific and Medical, Melbourne, Australia). Slides were covered with a coverslip (50-mm, No.1; Marienfeld) and inverted so that the protoplasts settled on the coverslip. All images were taken with a 40X water-immersion objective (NA = 1.2).

YFP and CFP fusion proteins were excited using the 514-nm and 458-nm lines of the Ar-laser respectively. Emission filters BP520–550 for YFP-tagged proteins and BP470–500 for CFP-tagged proteins were employed. Images showing the subcellular localization of YFP-DHH1, YFP-VCSc, DCP1-CFP and DCP2-CFP were captured in a single focal plane. To quantify P-body size and number, protoplasts isolated from transiently transformed tissue were imaged as a z-stack, with a 2-μm interval, capturing half the thickness of a cell. Co-localization studies were accomplished using the multi-tracking mode of LSM510 to ensure there was no cross-channel bleed through of fluorescence.

Images of P-bodies were analysed using ImageJ, a free tool available from the NIH (Abràmoff et al. 2004). Images were exported as TIFF files using the LSMS10AIM software and opened in Image J. Images were converted from RGB to 8-bit greyscale and calibrated by applying the “set scale” function after drawing a line over the scale bar. To quantify P-bodies, a threshold mask was set, allowing the regions in the image above a certain intensity to be “on”, while the rest of the image is “off”. Particle size was limited to those greater than 0.2 μm^2^ to ensure that noise was not counted as P-bodies. For one of the cells, the number of P-bodies was counted manually to check automated analysis settings were accurate. Entire z-stacks were loaded into the software and using the “analyse particles” function, the number of particles, circularity, area and perimeter of P-bodies was analysed.

## Electronic supplementary material

Figure S1 Co-localization analysis of VCS with DCP1. DCP1-CFP was co-expressed with YFP- VCSc in tobacco leaves. Blank arrowheads indicate the cytoplasmic foci marked by both proteins. While DCP1 frequently localised to cytoplasmic foci marked by VCSc, at times VCSc marked P-bodies devoid of DCP1 were observed as well. Solid white arrowheads indicate cytoplasmic foci marked by VCSc lacking DCP1. Bar =10 μm. (GIF 321 kb)

High Resolution Image (TIFF 563 kb)

Figure S2 Colocalization analysis of VCS with DCP2. DCP2-CFP was co-expressed with YFP-VCSc in tobacco leaves and then protoplasts isolated. P bodies marked with DCP2 when co-expressed with VCSc (solid arrowheads). Marked P-bodies devoid of DCP2 were observed as well (blank arrowheads). Bar =10 μm. (GIF 164 kb)

High Resolution Image (TIFF 204 kb)

Supplementary Video 1 Motion paths of P-bodies in transiently transformed tobacco epidermal leaf cells. P-bodies were visualised using YFP-VCSc. (AVI 39001 kb)

Supplementary Video 2 The actin cytoskeleton can be observed as fine filaments with P-bodies as round white spheres tracking the actin filaments in tobacco epidermal leaf cells. P-bodies and AFs were visualised simultaneously in tobacco epidermal cells co-transfected with YFP-VCSc (P-body marker) and GFP-faBD2 (AF label). (AVI 77400 kb)
