# Supplementary figures and images for: RNA processing body (P-body) dynamics in mesophyll protoplasts re-initiating cell division
Source: Protoplasma. 2016 Dec 7;254(4):1627–37. doi: 10.1007/s00709-016-1053-0 (PMC5487831; doi:10.1007/s00709-016-1053-0)

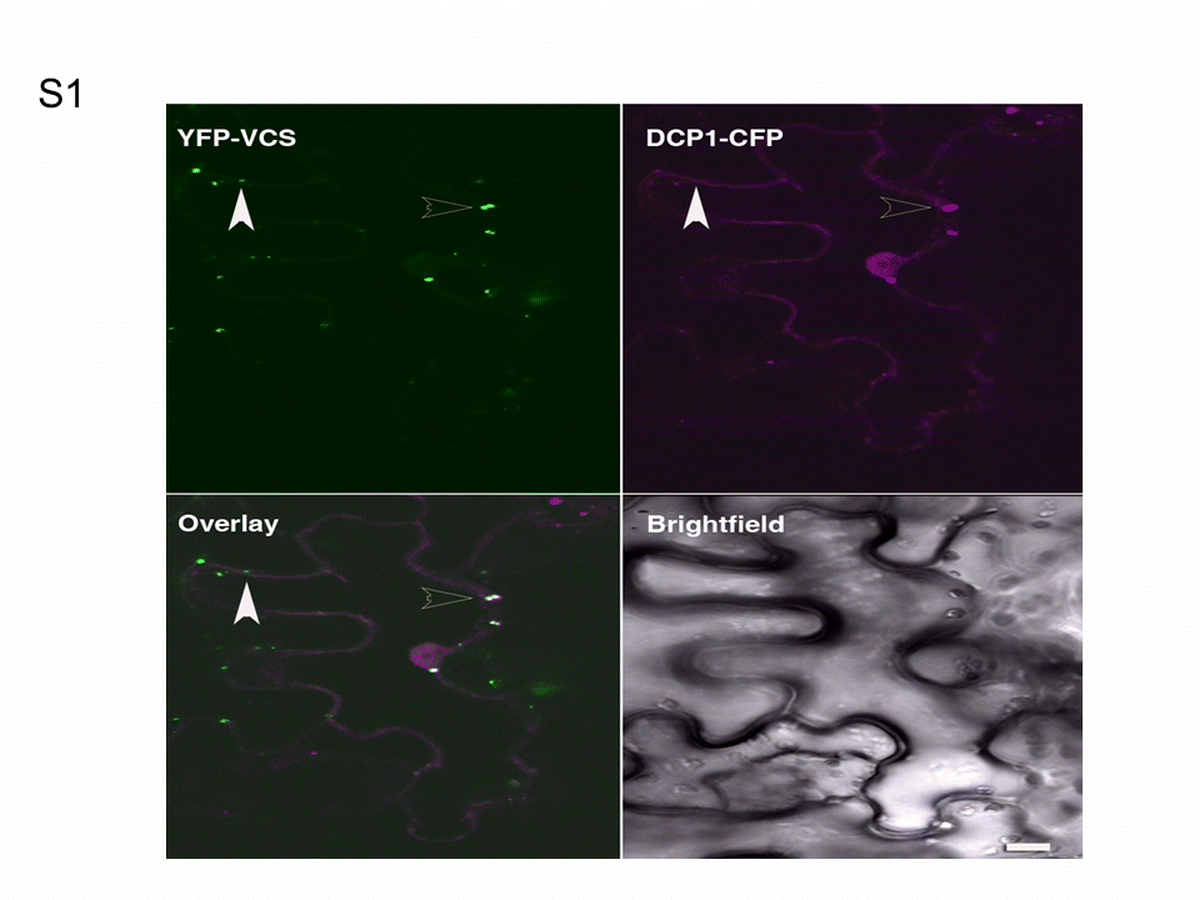

Supplement: Supplementary file 1 — Co-localization analysis of VCS with DCP1. DCP1-CFP was co-expressed with YFP- VCSc in tobacco leaves. Blank arrowheads indicate the cytoplasmic foci marked by both proteins. While DCP1 frequently localised to cytoplasmic foci marked by VCSc, at times VCSc marked P-bodies devoid of DCP1 were observed as well. Solid white arrowheads indicate cytoplasmic foci marked by VCSc lacking DCP1. Bar =10 μm. (GIF 321 kb) [file 709_2016_1053_Fig1_ESM.gif]

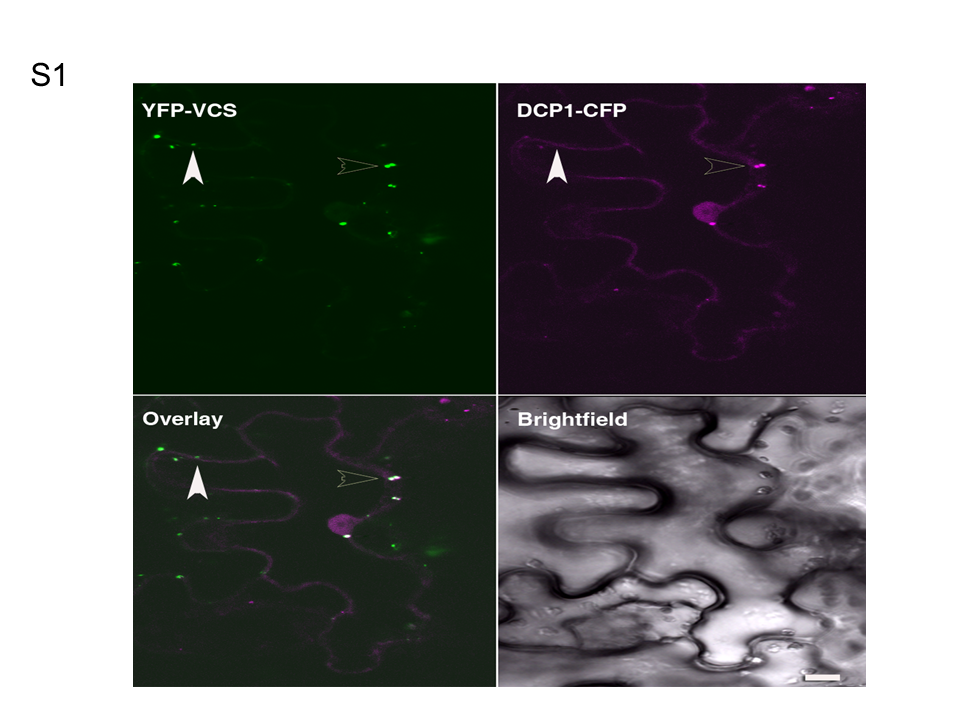

Supplement: Supplementary file 2 — High Resolution Image (TIFF 563 kb) [file 709_2016_1053_MOESM1_ESM.tif]

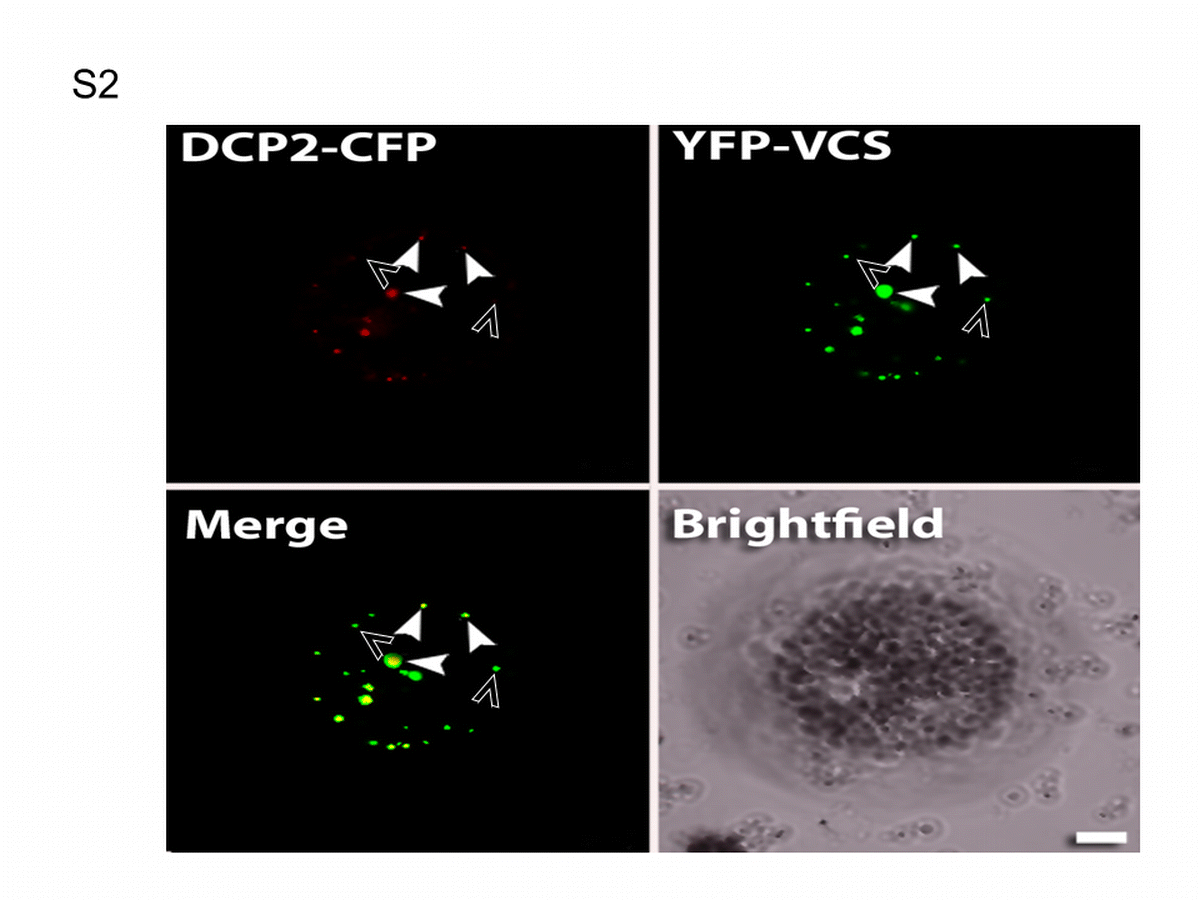

Supplement: Supplementary file 3 — Colocalization analysis of VCS with DCP2. DCP2-CFP was co-expressed with YFP-VCSc in tobacco leaves and then protoplasts isolated. P bodies marked with DCP2 when co-expressed with VCSc (solid arrowheads). Marked P-bodies devoid of DCP2 were observed as well (blank arrowheads). Bar =10 μm. (GIF 164 kb) [file 709_2016_1053_Fig2_ESM.gif]

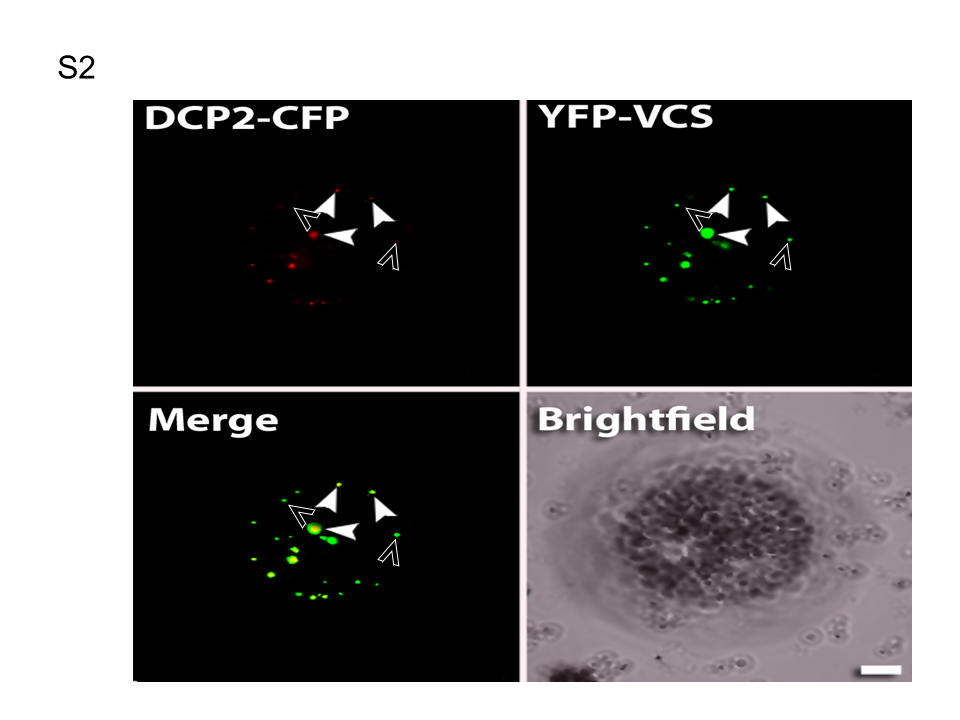

Supplement: Supplementary file 4 — High Resolution Image (TIFF 204 kb) [file 709_2016_1053_MOESM2_ESM.tif]
